# A case of late breast cancer metastases to both suprarenal glands 28 years after mastectomy

**DOI:** 10.1186/1897-4287-10-S3-A1

**Published:** 2012-04-20

**Authors:** T Banasiewicz, Ł Krokowicz, M Biczysko, M Janicka-Jedyńska, A Pławski, J Paszkowski, P Gronek, B Stawny, M Drews

**Affiliations:** 1 Department of General, Gastroenterological and Endocrinological Surgery, Poznan University of Medical Sciences, Poland; 2 Department of Clinical Pathology, Poznan University of Medical Sciences, Poland; 3 Institute of Human Genetics, Polish Academy of Sciences Poznan, Poland; 4 Department of Physiology, University School of Physical Education, Poznan, Poland

## 

We present an extremely rare case of late metastases of breast cancer to both suprarenal glands 28 years after mastectomy. The patient originally underwent Patey’s radical mastectomy of the left breast. In the follow-up, metastases were detected in the skin of the thorax and labia majora and were subsequently resected. During the most recent hospitalization metastases to both adrenal glands were detected (PET-CT, MRI) and removed. In genetic examination the entire coding sequences of BRCA1 and BRCA2 gene were screened for mutation by direct PCR product sequencing as described before by Gorski et al. No BRCA1 and BRCA2 gene mutations were found.

Histological examination revealed breast cancer metastasis to the adrenal glands. In our opinion our case can be the latest described metastases of breast cancer. The described case was an early stage of primary cancer (T1N0M0). Patients after breast cancer resection should still be followed up for late metastases. Late metastases including atypical sites should always be suspected regardless of time from initial presentation. The correlations between mutation and metastases of breast cancer needs to be discussed.

